# Comparison Between Topographic-Based and Manifest-Based Astigmatism Corrections in the Second (Visumax 800)-Generation Keratorefractive Lenticule Extraction Surgery: A Real-World Study

**DOI:** 10.3390/diagnostics15010098

**Published:** 2025-01-03

**Authors:** Chia-Yi Lee, Hung-Chi Chen, Shun-Fa Yang, Yi-Jen Hsueh, Chin-Te Huang, Jing-Yang Huang, Ie-Bin Lian, Chao-Kai Chang

**Affiliations:** 1 Institute of Medicine, Chung Shan Medical University, Taichung 40201, Taiwan; 2 Nobel Eye Institute, Taipei 10041, Taiwan; 3 Department of Ophthalmology, Jen-Ai Hospital Dali Branch, Taichung 41265, Taiwan; 4 Department of Ophthalmology, Chang Gung Memorial Hospital, Linkou, Taoyuan 33305, Taiwan; 5 Center for Tissue Engineering, Chang Gung Memorial Hospital, Linkou, Taoyuan 33305, Taiwan; 6 Department of Medicine, Chang Gung University College of Medicine, Taoyuan 33305, Taiwan; 7 Department of Medical Research, Chung Shan Medical University Hospital, Taichung 40201, Taiwan; 8 Department of Ophthalmology, Chung Shan Medical University Hospital, Taichung 40201, Taiwan; 9 Department of Ophthalmology, School of Medicine, Chung Shan Medical University, Taichung 40201, Taiwan; 10 Institute of Statistical and Information Science, National Changhua University of Education, Chunghua 50007, Taiwan; 11 Department of Optometry, Da-Yeh University, Chunghua 51591, Taiwan

**Keywords:** keratorefractive lenticule extraction, visumax 800, smile pro, uncorrected distance visual acuity, astigmatism

## Abstract

**Objectives**: To evaluate the effectiveness of astigmatism correction between topographic- and manifest-based methods in individuals who underwent second-generation keratorefractive lenticule extraction (KLEx) surgery. **Methods**: This study was conducted with participants who underwent second-generation KLEx surgery. After exclusion, there were 46 and 43 participants in the manifest and topographic groups, respectively. The main outcomes were postoperative uncorrected distance visual acuity (UDVA), spherical equivalent (SE), and residual astigmatism. The independent T-test and generalized estimate equation were used to investigate differences between the two groups. **Results**: Three months postoperatively, UDVA was 0.02 ± 0.04 in the manifest group and 0.00 ± 0.06 in the topographic group which also revealed no significant difference (*p* = 0.155). Also, the SE value in the two groups three months postoperatively was statistically similar (−0.57 ± 0.48D versus −0.63 ± 0.62D, *p* = 0.574). The final residual astigmatism was −0.26 ± 0.27 in the topographic group which was significantly lower than the −0.51 ± 0.40 in the manifest group (*p* < 0.001). Moreover, the amplitude of astigmatism change was significantly lower in the topographic group (*p* = 0.002). In the subgroup analysis, UDVA and residual astigmatism were significantly better in the topographic group than in the manifest group (both *p* < 0.05). **Conclusions**: The topographic-based method represents a better astigmatism correction than the manifest-based method in second-generation KLEx surgery, especially in the low astigmatism population.

## 1. Introduction

Keratorefractive surgeries are a type of surgery that reduces the degree of myopia, astigmatism, and hyperopia and have been developed over the last few decades [1,2]. Laser in situ keratomileusis and photorefractive keratectomy are two types of keratorefractive surgeries that were first introduced between 1980 and 1990 in peer-reviewed journals and have produced fair surgical outcomes [2]. Some postoperative complications can occur in the two keratorefractive surgeries, including eye pain, corneal abrasion, and dry eye disease [3]. Nevertheless, fair postoperative uncorrected distance visual acuity (UDVA) of 20/20 or better has been found in more than 70 percent of individuals who underwent laser in situ keratomileusis and photorefractive keratectomy [4,5].

Recently, keratorefractive lenticule extraction (KLEx) has been introduced in the field of refractive surgery, previously known as small incision lenticule extraction [6]. During the KLEx surgery, a corneal lenticule was created using a femtosecond laser, and the surgeon removed it to correct myopia and astigmatism [7,8,9]. Compared to keratorefractive surgeries invented earlier, under-correction of astigmatism than the laser in situ keratomileusis has been observed in first-generation KLEx surgery [10]. In terms of advantages, KLEx surgery is associated with similar postoperative visual acuity and a lower risk of postoperative dry eye disease [11,12,13,14,15,16].

Last year, second-generation KLEx surgery was conducted under clinical conditions [17]. Second-generation KLEx surgery is superior to first-generation KLEx surgery in terms of laser strike velocity and the eye-tracking system [18,19]. However, the ultimate method of astigmatism correction for second-generation KLEx surgery has not yet been determined. Although the eye-tracking system may increase the accuracy of astigmatism correction, whether different methods would alter the predictability of astigmatism correction in second-generation KLEx surgery has not been fully elucidated.

Therefore, the aim of the presented study was to evaluate the visual and refractive results of second-generation KLEx surgeries with topographic-based and manifest-based astigmatism correction. The accuracy of astigmatism correction in the low- and high-astigmatism populations was also investigated.

## 2. Materials and Methods

### 2.1. Individual Selection

This study was conducted at the Nobel Eye Institute, which has more than 15 clinics in the Northern, Central and Southern regions of Taiwan. The participants were enrolled according to the following inclusion criteria: (1) age ranged from 20 to 55 years; (2) presented with cycloplegia sphere power lower than −1.00 diopter (D) but higher than −9.00D; (3) cycloplegia cylinder power from +0.00D to −5.00D; (4) received second-generation KLEx surgery in the Nobel Eye Institute; and (5) followed-up in the Nobel Eye institute after the second-generation KLEx surgery longer than three months. On the other hand, the following exclusion criteria were utilized in the present study to exclude those with significant ocular morbidities: (1) best-corrected visual acuity (BCVA) worse than 20/100; (2) pre-existing severe ocular diseases involving central corneal opacity, proliferative diabetic retinopathy, eyeball rupture, keratoconus, end-stage glaucoma, macula-off retinal detachment, persistent uveitis, and central retinal venous occlusion; (3) change in spherical equivalent (SE) for more than 0.50D during the previous year; and (5) pregnancy or breastfeeding status in the previous three months. We decided whether the eye would receive topographic-based and manifest-based astigmatism corrections by drawing lots, and only the right eye of each participant was involved in the present study. Finally, a total number of 65 and 71 eyes were categorized into topographic and manifest groups, respectively.

### 2.2. Surgery Information

All second-generation KLEx surgeries in the present study were performed by two experienced refractive specialists (C.-Y.L. and C.-K.C.) with a second-generation femtosecond laser device (Visumax 800; Carl Zeiss, Göschwitzer Str., Jena, Germany). Regarding the astigmatism correction method, the correction of astigmatism in the manifest group primarily based on both the subjective refractions with and without cycloplegia, and the cycloplegic autorefractometry value served as assistance. In contrast, the topographic method uses the corneal cylinder power in the topographic image as the astigmatism correction amount. If the difference between manifest astigmatism and topographic astigmatism was greater than −0.50D, the average values of manifest and topographic astigmatism were applied. The surgical procedure is similar to the approach described in our previous publications [20,21]. After evaluating the mesopic pupil size and ablation depth, the optic zone of each eye was built from 5.5 to 6.9 mm. In addition, the corneal incision site was made at 105 degrees with a length of 3.0 mm. The angle kappa was determined by the doctor according to the coaxial sighted corneal light reflex approach and the topographic appearance. The angle kappa obtained from optical biometry (IOL Master 700, Carl Zeiss, Göschwitzer Str., Jena, Germany) was also represented by the Visumax 800 software. After the confirmation of angle kappa via the above two approaches, the corneal surface was settled in the suction ring and the Visumax 800 device started femtosecond laser release. After the above laser treatment, the ophthalmologist took a distinctive spatula to divide the upper and lower corneal lenticule surfaces, and the ophthalmologist derived the corneal lenticule via forceps. The levofloxacin solution and prednisolone solution were administered for one week postoperatively, and then the medications were changed to sulfamethoxazole solution and fluorometholone solution for three weeks. Moreover, the hyaluronic acid-containing artificial tears were instilled for approximately two months.

### 2.3. Ocular Examination

All participants underwent second-generation KLEx surgery received same preoperative and postoperative examinations in all clinics of the Nobel Eye Institute. Before the second-generation KLEx surgery, manifest BCVA, cyclopegia refraction, steep keratometry (K), flat K, corneal astigmatism, central corneal thickness (CCT) at the apex and thinnest region, angle kappa, and pupil diameter were examined using the Snellen chart, autorefractor (KR-8900, Topcon, Itabashi-ku, Tokyo, Japan), topographic machine (TMS-5, Tomey Corporation, Nagoya, Aichi, Japan), and biometry machine (IOL Master 700, Carl Zeiss, Göschwitzer Str., Jena, Germany). It is worth noting that the SE was set as sphere power plus half of cylinder power and the angle kappa number was determined as the average angle kappa amount from topographic and biometric devices in the present study. Regarding surgical data, the optic zone (OZ), cap thickness, lenticule thickness, side-cut depth, and the residual stromal thickness (RST) were recorded for all the eyes. After the second-generation KLEx surgery, the UDVA, sphere power, and cylinder power were measured in each eye. The same devices were used for both the preoperative evaluation and the postoperative evaluation for all participants. The examination results before, one day after, one week after, one month after, and three months after the second-generation KLEx surgery were used in the consecutive analyses.

### 2.4. Statistical Analysis

The SPSS version 20.0 (SPSS Inc., Chicago, IL, USA) was used for our statistical analysis. The Shapiro–Wilk test was used to confirm the normality of our study population that received the second-generation KLEx surgery. After the analysis, the normal distribution for our study population was concluded (*p* > 0.05). A descriptive analysis represented the distribution of demographic and ocular data involving sex, age, refraction, surgical information, and topographic information between the two groups. For the comparison between baseline characters between the topographic and manifest groups, an independent T-test was employed. In the next step, the independent T-test was used to weigh the efficiency (or the UDVA), predictability (or the SE), and the residual astigmatism between the topographic and manifest groups after the performance of surgery. The bar chart was conducted to reveal the percentage who accomplished UDVA of 20/20, SE within ±1.00D, and residual astigmatism within± 0.50D in the topographic and manifest groups. The generalized estimate equation was used to check the trend of UDVA, SE, and residual astigmatism changes in different groups and produce the adjusted odds ratio (aOR) with an associated 95% confidence interval (CI) between the topographic and manifest groups after adjustment for age, sex, preoperative BCVA, and preoperative refraction. The single-angle plot was drawn to further present the refractive astigmatism correction in the two groups. The two groups were then stratified based on the degree of preoperative astigmatism (<−1.50D as low astigmatism population and over −1.50D as high astigmatism population), and the differences in UDVA, SE, and residual astigmatism at the final visit (i.e., three months after the surgery) were compared between different subgroups via the Mann–Whitney U test. Statistical significance was set at *p* < 0.05, and a *p* value < 0.001 was represented as *p* < 0.001 in the present study.

## 3. Results

The initial characteristics of the topographic and manifest groups are represented in Table 1. The mean age was 34.39 ± 9.98 years and 31.74 ± 5.62 years in the manifest and topographic groups, respectively. The difference in mean age between the groups did not reach statistical significance (*p* = 0.124). Both the sex and laterality distribution also revealed non-significant differences between the topographic group and manifest group (both *p* > 0.05), whereas the number of systemic diseases was significantly higher in the manifest group than the topographic group (*p* = 0.045). Regarding the refractive, topographic, and surgical indices, the topographic group revealed a significantly larger cap diameter and thicker cap (both *p* > 0.05), while no significant difference in other indices between the two groups was found (all *p* > 0.05) (Table 1).

**Figure 1:**
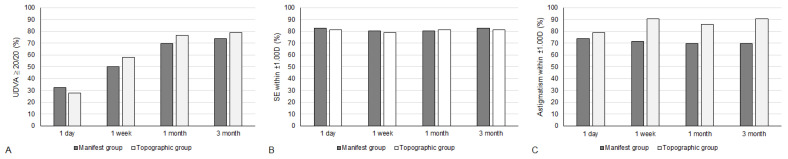
The percentage of participants within specific range of visual and refractive outcomes between the two groups. (**A**) The percentage of participants that reached an uncorrected distance visual acuity of 20/20; (**B**) The percentage of participants that reached spherical equivalent within ±1.00 diopter; (**C**) The percentage of participants that reached residual astigmatism within ±0.50 diopter.

**Figure 2:**
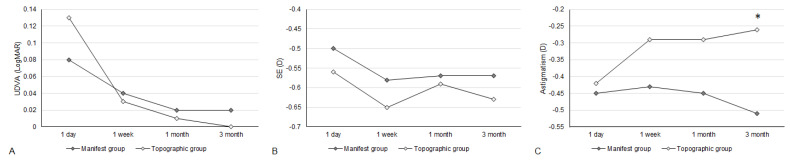
The trend of visual and refractive parameters between the two groups. (**A**) The trend of uncorrected distance visual acuity alteration; (**B**) The trend of spherical equivalent alteration; (**C**) The trend of residual astigmatism alteration. * Significant difference between groups.

**Figure 3:**
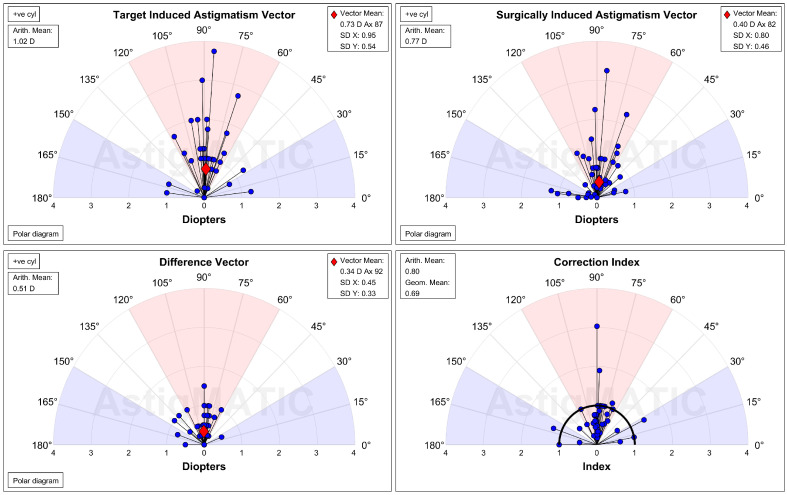
The single-angle plot for the refractive astigmatism in the manifest group. D: diopter, SD: standard deviation.

**Figure 4:**
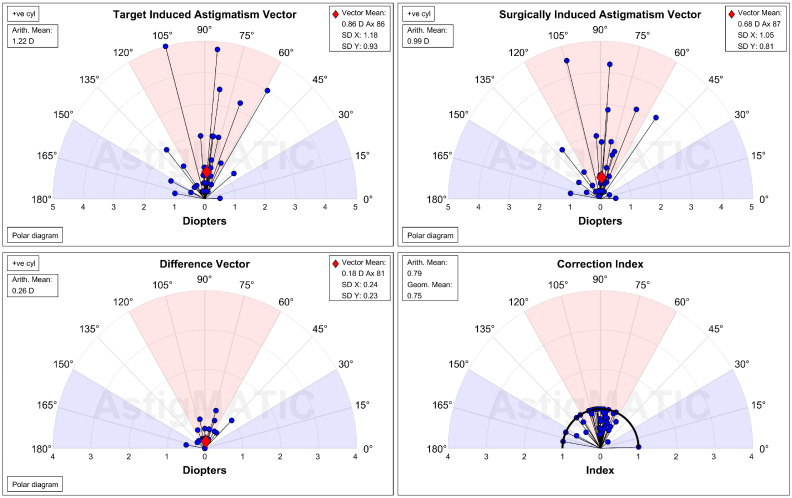
The single-angle plot for the refractive astigmatism in the topographic group. D: diopter, SD: standard deviation.

The UDVA one day after the second-generation KLEx surgery was 0.08 ± 0.09 in the manifest group and 0.13 ± 0.15 in the topographic group, and the difference between them was statistically insignificant (*p* = 0.077). Three months postoperatively, the UDVA was 0.02 ± 0.04 in the manifest group and 0.00 ± 0.06 in the topographic group which also revealed no significant difference (*p* = 0.155) (Table 2). For SE, the initial postoperative SE was −0.50 ± 0.50D in the manifest group and −0.56 ± 0.59D in the topographic group without significant difference (*p* = 0.589), and the SE value was −0.57 ± 0.48D in the manifest group and −0.63 ± 0.62D in the topographic group three months postoperatively which was statistically similar (*p* = 0.574) (Table 2). The residual astigmatism one day postoperatively was −0.42 ± 0.36D in the topographic group which was not significantly lower than the −0.45 ± 0.33D in the manifest group (*p* = 0.592), and the final residual astigmatism was −0.26 ± 0.27 in the topographic group which was significantly lower than the −0.51 ± 0.40 in the manifest group (*p* < 0.001) (Table 2). As shown in Figure 1A–C, the percentage of UDVA reached 20/20, SE within ± 1.00D, and residual astigmatism within ± 0.50D in the two groups. On the other hand, the trend of UDVA (aOR: 1.006, 95% CI: 0.985–1.028, *p* = 0.563) and SE (aOR: 0.923, 95% CI: 0.749–1.139, *p* = 0.455) did not show a significant difference between the two groups, while the change in astigmatism demonstrated a significantly lower amplitude in the topographic group than in the manifest group (aOR: 1.179, 95% CI: 1.061–1.310, *p* = 0.002) (Figure 2A–C). The single-angle plot for the manifest group and topographic group are demonstrated in Figure 3 and Figure 4, respectively.

In the subgroup analysis, UDVA, SE, and residual astigmatism showed no significant difference between the two groups with high astigmatism (all *p* > 0.05) (Table 3). The SE in the low astigmatism population was also similar between the two groups (*p* = 0.647) (Table 3). On the other hand, UDVA was significantly better in the topographic group than in the manifest group in patients with low astigmatism (*p* = 0.019), and residual astigmatism was also significantly lower in the topographic group than in the manifest group in the low astigmatism population (*p* = 0.001) (Table 3).

## 4. Discussion

In short, second-generation KLEx surgery with topographic-based astigmatism correction demonstrated similar postoperative UDVA and SE but significantly lower residual astigmatism than manifest-based astigmatism correction. Moreover, the degree of astigmatism progressively reduced in the topographic-based astigmatism correction group. In contrast, topographic-based astigmatism correction presented better UDVA and residual astigmatism than manifest-based astigmatism correction in the low astigmatism population.

The topographic and manifest groups showed similar postoperative UDVA values throughout the study period. Concerning UDVA in keratorefractive surgeries, both the first- and second-generation KLEx surgeries demonstrated a mean UDVA better than 20/25 after a follow-up period of three months or more [7,18]. In addition, more than 80 percent of patients reached a UDVA of 20/20 after first- and second-generation KLEx surgeries [7,18]. However, it is uncertain whether different methods of astigmatism correction would contribute to significant differences in the postoperative UDVA of second-generation KLEx surgery. To the best of our knowledge, these results may be a foundation for similar efficiency of second-generation KLEx surgery with topographic-based or manifest-based astigmatism correction. Moreover, the ophthalmic characteristics in the topographic and manifest groups were largely similar, except for cap thickness and diameter. Consequently, the homogeneity of the two groups may be adequate, which could elevate the integrity of the present study. The trend of UDVA change showed numerically worse UDVA initially in the topographic group and numerically better UDVA in the topographic group. A possible explanation is that the topographic-based method removes a larger proportion of corneal tissue because it plans a higher degree of astigmatism correction. Consequently, visual recovery was slower in the initial period because of the high degree of refractive correction associated with delayed recovery [22]. Despite the relatively worse initial UDVA, the mean UDVA, and the ratio to reach UDVA 20/20 in the topographic group were not significantly better than those in the manifest group after three months. These results confirmed the efficiency of the two methods.

The postoperative predictability regarding the mean SE showed no significant difference between the topographic and manifest groups in the present study. In a previous study, the postoperative SE was around −0.10 to −0.20D in individuals who underwent first-generation KLEx surgery [23]. In addition, the postoperative SE after first-generation KLEx surgery showed a larger amplitude in the high astigmatism population which was from −1.63D to +1.38D [23]. In the research that discussed the predictability of second-generation KLEx surgery, more than 80 percent of patients presented with SE within ±0.50D a few months after the surgery [18]. More than 80 percent of patients achieved an SE within ±1.00D in both the topographic and manifest groups, which indicates the fair predictability of both astigmatism correction methods. The absolute value of SE was higher in the topographic group than in the manifest group, but the difference was about −0.05D which is clinically insignificant. In addition, the trends of SE alteration were similar between the two groups, indicating similar overall refractive stability. On the other hand, postoperative residual astigmatism was significantly less in the topographic group than in the manifest group starting from one month postoperatively. The mean postoperative residual astigmatism in the topographic group was only half that in both the manifest group and previous studies that surveyed first-generation KLEx surgery [24,25,26]. Because first-generation KLEx surgery tends to undercorrected astigmatism [10], adjustment of astigmatism correction by topography in KLEx surgery may be needed. Furthermore, 90 percent of participants in the topographic group illustrated a residual astigmatism within ±0.50D compared to the 70 percent in the manifest group, and the trend of astigmatism in the topographic group was significantly lower than that in the manifest group. The above evidence may indicate that topographic-based astigmatism correction is a superior approach for the general population who have undergone second-generation KLEx surgery.

Concerning the efficiency, predictability, and postoperative residual astigmatism in the participants with different preoperative astigmatism extensions, the final UDVA, SE, and postoperative residual astigmatism between the topographic and manifest groups demonstrated insignificant differences, indicating that the outcomes between the two methods were similar in patients with high astigmatism. On the other hand, the topographic group presented with a better UDVA and lower postoperative residual astigmatism at the last follow-up than the manifest group in the low astigmatism population. However, only a few studies have reported this phenomenon. Although the difference in UDVA between the two subgroups was less than one Snellen line, the difference in residual astigmatism between the two subgroups was more than −0.25D. Considering the preoperative mean cylinder power was approximately −0.70D in the low astigmatism population, the difference in postoperative residual astigmatism between the two subgroups may be prominent. The different effects of topographic-based astigmatism correction on low and high astigmatism populations may be explained by the fact that patients with high astigmatism are harder to correct. In a previous study, high astigmatism was associated with higher postoperative residual myopia and astigmatism in different keratorefractive surgeries [27,28]. Moreover, the incidence was four-fold higher in the population receiving laser in situ keratomileusis with high astigmatism [29]. Consequently, topographic-based astigmatism correction in patients with high astigmatism may not show the same accuracy as in the low astigmatism population. However, the postoperative residual astigmatism in the high astigmatism population was numerically lower in the topographic group with an amplitude of −0.15D, which might imply that the topographic-based method has an advantage over the manifest-based method, at least to some extent.

Regarding the postoperative parameters between the present study and the results of keratorefractive surgeries in previous publications, the mean UDVA in the present study was 0.01 after the entire follow-up period, and another study performed laser in situ keratomileusis demonstrated similar results concerning UDVA numbers [30]. When comparing to the photorefractive keratectomy, the mean UDVA numbers of second-generation KLEx surgery in our research were similar to the mean UDVA numbers of an earlier article evaluating photorefractive keratectomy [5]. In the case of the predictability of different refractive surgeries, approximately 82 percent of participants exhibited an SE within ±1.00D after the surgery in the present study, and the results of our participants are comparable to the earlier articles conducting laser in situ keratomileusis and the first-generation KLEx surgery [23,30]. For the cylinder power, the mean final residual astigmatism in the topographic group postoperatively read −0.26D, which was comparable to the results in earlier articles that investigating the first- and second-generation KLEx surgeries [31,32]. In addition, the remaining astigmatism in the high astigmatism subgroup with the topographic-based correction method was numerically lower than the remaining astigmatism of both laser in situ keratomileusis and transepithelial photorefractive keratectomy [33]. The above evidence may support the satisfactory surgical quality of second-generation KLEx surgery in our establishment.

However, the present study had some obvious limitations. First, the second-generation KLEx surgeries in the present study were performed by two different surgeons. Although the two physicians are highly experienced and operated under identical protocols for second-generation KLEx surgery, the difference in manipulation may still affect the results to some extent. Secondly, the participant numbers were relatively depressed, only 89 eyes were comprised in the present study. The depressed participant numbers may have contributed to statistical partiality. Furthermore, all participants in the present study were Han Taiwanese; thus, the extraneous validity of the present study could be trimmed. Also, we did not perform the postoperative corneal irregularity examination like the corneal higher-order aberration and angle kappa measurement, which is a crucial point for surveying the etiology of better astigmatism correction in the topographic group. Finally, the degree of preoperative myopia between the two groups was slightly different, which may affect the statistical analyses and the results. Notwithstanding, the difference in myopia between the topographic and manifest groups was fewer than −0.50D, and might not have a compelling effect on the analysis outcome.

## 5. Conclusions

In conclusion, topographic-based astigmatism correction contributes to lower postoperative astigmatism than manifest-based astigmatism correction in second-generation KLEx surgeries. Furthermore, this phenomenon was more prominent in the participants with low astigmatism. Consequently, topographic-based astigmatism correction could be recommended for individuals scheduled for second-generation KLEx surgery, especially in those with a low astigmatism degree. Further large-scale prospective studies to investigate the effectiveness of topographic-based astigmatism correction in individuals with different astigmatism patterns are mandatory.

## Figures and Tables

**Table 1:** The baseline characters of the study population.

Character	Manifest Group (N: 46)	Topographic Group (N: 43)	*p*
Age (mean ± SD)	34.39 ± 9.98	31.74 ± 5.62	0.124
Sex (male: female)	22:24	14:29	0.195
Laterality (right: left)	31:15	22:21	0.135
Disease			0.045 *
Hypertension	5	0	
Diabetes mellitus	2	0	
Other	4	2	
BCVA (LogMAR)	0.00 ± 0.00	0.00 ± 0.02	0.160
Manifest refraction			
Sphere	−4.84 ± 1.73	−5.62 ± 2.06	0.056
Cylinder	−0.97 ± 0.78	−1.11 ± 1.25	0.524
SE	−5.33 ± 1.93	−6.19 ± 2.37	0.061
Cycloplegia refraction			
Sphere	−4.75 ± 1.71	−5.27 ± 2.62	0.271
Cylinder	−1.02 ± 0.82	−1.21 ± 1.22	0.401
SE	−5.26 ± 1.93	−5.88 ± 2.91	0.245
Topography			
Steep K	43.87 ± 1.77	43.32 ± 1.74	0.146
Flat K	42.52 ± 1.43	41.95 ± 1.51	0.071
Cylinder power	1.32 ± 0.59	1.43 ± 0.87	0.500
CCT at apex	558.83 ± 32.46	554.81 ± 42.89	0.619
CCT at Thinnest	552.54 ± 32.57	549.07 ± 42.44	0.665
Angle Kappa	0.19 ± 0.09	0.19 ± 0.09	0.805
Pupil diameter	3.64 ± 0.42	3.76 ± 0.67	0.340
Schirmer test	16.54 ± 8.47	15.36 ± 7.18	0.481
Optic zone	6.49 ± 0.05	6.55 ± 0.20	0.062
Side-cut depth	15.98 ± 4.55	16.00 ± 6.79	0.986
Cap diameter	7.49 ± 0.05	7.57 ± 0.22	0.024 *
Cap thickness	117.83 ± 5.54	115.12 ± 7.03	0.045 *
RST	325.13 ± 36.87	312.33 ± 26.76	0.063
Lenticule thickness	114.24 ± 30.44	127.47 ± 36.06	0.064

BCVA, best-corrected visual acuity; CCT, central corneal thickness; KLEx, keratorefractive lenticule extraction; N, number; RST, residual stromal thickness; SD, standard deviation; SE, spherical equivalent. * Significant difference between the two groups.

**Table 2:** Postoperative visual and refractive condition between the two groups.

Outcome	Manifest Group (N: 46)	Topographic Group (N: 43)	*p*
UDVA (mean ± SD)			
1 day	0.08 ± 0.09	0.13 ± 0.15	0.077
1 week	0.04 ± 0.06	0.03 ± 0.08	0.738
1 month	0.02 ± 0.04	0.01 ± 0.05	0.291
3 months	0.02 ± 0.04	0.00 ± 0.06	0.155
SE (mean ± SD)			
1 day	−0.50 ± 0.50	−0.56 ± 0.59	0.589
1 week	−0.58 ± 0.56	−0.65 ± 0.71	0.593
1 month	−0.57 ± 0.50	−0.59 ± 0.62	0.871
3 months	−0.57 ± 0.48	−0.63 ± 0.62	0.574
Cylinder (mean ± SD)			
1 day	−0.45 ± 0.33	−0.42 ± 0.36	0.592
1 week	−0.43 ± 0.47	−0.29 ± 0.26	0.087
1 month	−0.45 ± 0.37	−0.29 ± 0.29	0.026 *
3 months	−0.51 ± 0.40	−0.26 ± 0.27	<0.001 *

KLEx, keratorefractive lenticule extraction; N, number; SD, standard deviation; SE, spherical equivalent; UDVA, uncorrected distance visual acuity. * Significant difference between groups.

**Table 3:** Visual and refractive conditions three months after the keratorefractive lenticule extraction with different astigmatism degrees.

Outcome	Manifest Group	Topographic Group	*p*
High astigmatism			
UDVA	0.02 ± 0.05	0.03 ± 0.09	0.719
SE	−0.65 ± 0.44	−0.61 ± 0.42	0.786
Cylinder	−0.63 ± 0.46	−0.50 ± 0.27	0.433
Low astigmatism			
UDVA	0.02 ± 0.04	0.00 ± 0.04	0.019 *
SE	−0.54 ± 0.49	−0.64 ± 0.68	0.647
Cylinder	−0.46 ± 0.38	−0.17 ± 0.21	0.001 *

KLEx, keratorefractive lenticule extraction; N, number; SE, spherical equivalent; UDVA, uncorrected distance visual acuity. * Significant difference between groups.

## Data Availability

The data used in this study are available from the corresponding author upon reasonable request.
